# Downstream and Intermediate Interactions of Synovial Sarcoma-Associated Fusion Oncoproteins and Their Implication for Targeted Therapy

**DOI:** 10.1155/2012/249219

**Published:** 2012-03-25

**Authors:** Joanna Przybyl, Monika Jurkowska, Piotr Rutkowski, Maria Debiec-Rychter, Janusz A. Siedlecki

**Affiliations:** ^1^Department of Molecular Biology, The Maria Sklodowska-Curie Memorial Cancer Centre and Institute of Oncology, 02-781 Warsaw, Poland; ^2^Postgraduate School of Molecular Medicine, Warsaw Medical University, 02-091 Warsaw, Poland; ^3^Doctoral School of Biomedical Sciences, Catholic University of Leuven, 3000 Leuven, Belgium; ^4^Department of Molecular Diagnostics Laboratory, The Maria Sklodowska-Curie Memorial Cancer Centre and Institute of Oncology, 02-781 Warsaw, Poland; ^5^Department of Soft Tissue/Bone Sarcoma and Melanoma, The Maria Sklodowska-Curie Memorial Cancer Centre and Institute of Oncology, 02-781 Warsaw, Poland; ^6^Department of Human Genetics, Catholic University of Leuven, 3000 Leuven, Belgium

## Abstract

Synovial sarcoma (SS), an aggressive type of soft tissue tumor, occurs mostly in adolescents and young adults. The origin and molecular mechanism of the development of SS remain only partially known. Over 90% of SS cases are characterized by the t(X;18)(p11.2;q11.2) translocation, which results mainly in the formation of *SS18-SSX1* or *SS18-SSX2* fusion genes. In recent years, several reports describing direct and indirect interactions of *SS18-SSX1/SSX2* oncoproteins have been published. These reports suggest that the fusion proteins particularly affect the cell growth, cell proliferation, TP53 pathway, and chromatin remodeling mechanisms, contributing to SS oncogenesis. Additional research efforts are required to fully explore the protein-protein interactions of SS18-SSX oncoproteins and the pathways that are regulated by these partnerships for the development of effective targeted therapy.

## 1. Introduction

Synovial sarcoma (SS) represents approximately 10% of soft tissue sarcomas. SS is an aggressive type of tumor, which originates mainly in the extremities but may occur at any anatomic site. Approximately 50% of SS patients develop metastases, which mainly occur in the lungs. Contrary to the “synovial sarcoma” term, SS has no biological or pathological relation to synovium [1, 2]. SS is considered as a sarcoma of unknown origin, but recent findings have pointed to either a neural [3], myogenic [4], or multipotent mesenchymal stem cell [5, 6]. SS occurs in patients at any age but mainly in adolescents and young adults, and it occurs more commonly in males. A single SS case in a human fetus has been reported by Duband et al. [7]. Considering histology, SS is either biphasic or monophasic. Biphasic SS has epithelial and spindle cell components in varying proportions [1, 8–11]. The epithelial cells form glands with lumina or papillary structures. The spindle cell component often exists as a monophasic SS, in which spindle cells typically form dense cellular sheets or fascicles. Another rare variant of SS, which is classified as poorly differentiated, is characterized by ovoid or rounded small cells similar to cells in other small round cell tumors. This histological type is associated with the worst clinical outcome [1, 11, 12]. Well-established immunohistochemical markers of SS include epithelial membrane antigen (EMA), transducin-like enhancer protein 1 (TLE1), and cytokeratins (CK7, CK19 and pan-cytokeratin) [13–18]. Immunohistochemical analysis is especially important in the differential diagnosis of monophasic and poorly differentiated SS. However, these markers are not specific enough, and the final SS diagnosis is currently frequently supported by cytogenetic or molecular tests. 

The first description of *t*(*X*;18) and the report linking SS with the nonrandom presence of *t*(*X*;18) were published in 1986 [19, 20]. Since 1986, numerous studies have been undertaken to investigate the role of the *t*(*X*;18)(*p*11.2;*q*11.2) translocation in SS development and maintenance. The characteristic fusion gene found in more than 90% of SS cases involves the *SS18 *(previously known as *SYT*) gene on chromosome 18 and one of the *SSX* genes on the X chromosome [9, 10, 21]. Nine *SSX* genes (*SSX1-9*) have been described, and they are highly homologous. The nucleotide sequence homology of these genes ranges from 87 to 96%, and the amino acid sequence homology ranges from 73 to 92%. The SSX gene family also includes 10 pseudogenes, all of which are located on the X chromosome [22]. The SS18 protein, SSX protein, and SS18-SSX fusion protein are localized in nucleus [23, 24].

## 2. Function of *SS18* and *SSX* Genes

**Figure 1:**
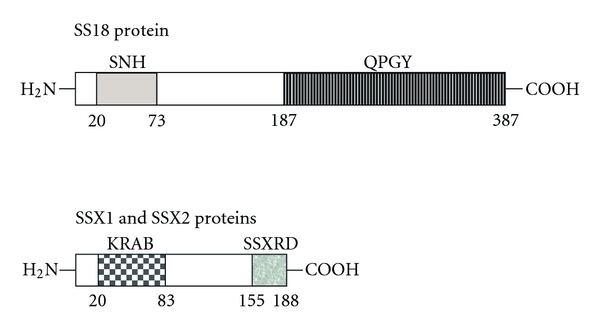
Schematic diagram shows the domains structure of SS18 and SSX proteins. The amino acid residues representing the boundaries of selected domains are indicated. SNH-SS18 N-terminal domain; QPGY-SS18 domain rich in glutamine, proline, glycine, and tyrosine; KRAB-SSX Krüppel-associated box domain; SSXRD-SSX transcriptional repression domain.

*SS18* is a ubiquitously expressed gene that encodes a 387-amino acid protein. This protein has two functional domains, and both domains are involved in the regulation of transcription. The SNH domain at the N-terminus is localized between amino acid residues 20 and 73, and it is likely involved in the inhibition of transcriptional activation. The second domain is called the QPGY domain, and it is rich in glutamine, proline, glycine, and tyrosine residues. This domain is localized between amino acid residues 187 and 387, and it has been described as a transcriptional activator (Figure 1). The ribonucleoprotein, SYT-interacting protein/coactivator activator (SIP/CoAA), specifically binds to the QPGY domain of SS18. SIP/CoAA is an RNA splicing modulator and coactivator of transcription, which may point to a hypothetical mechanism of transcriptional regulation by SS18 and the SS18-SSX fusion transcript [25, 26]. Furthermore, the SS18 protein interacts with P300 (a transcriptional coactivator and histone acetyltransferase), which results in the formation of a P300/SS18 complex that regulates cell adhesion [27]. Accordingly, Kimura and coworkers [28] suggested that SS18 may control the expression of P300. Moreover, SS18 has been shown to interact with the following proteins: the leukemia-associated protein, AF10 (also known as MLLT10) [29]; components of the chromatin remodeling complex, including hBRM and BRG1 (also known as SMARCA2 and SMARCA4, resp.) [30, 31] and the growth factor receptor-bound protein, GBR2 [32]. Using mouse cDNA assays for the systematic analysis of protein/protein interactions, Suzuki et al. [33] demonstrated that SS18 interacts with the H3.3A histone. Ito and colleagues [34] reported that the SS18 protein interacts with mSIN3A (a component of histone deacetylase complex), resulting in mSIN3A repression of transcriptional activity mediated by SS18. Kato and coworkers [35] showed that SS18 associates also with the human SNF/SWI complex, which is a chromatin remodeling factor. Moreover, SS18 has an important role in embryonic development because it participates in the regulation of cell motility and cytoskeletal organization [28]. Furthermore, SS18 affects the expression of genes important for placental development, such as peroxisome proliferator-activated receptor-binding protein (*PPARB*) [36]. The SS18 protein may also have an essential role in epithelial morphogenesis [37].

The *SSX1* and *SSX2* genes encode proteins consisting of 188 amino acid residues. The *SSX* genes are normally expressed in the testis and thyroid, and these genes are also expressed in numerous types of human cancers including melanoma [38–40], multiple myeloma [41, 42], non-Hodgkin's lymphoma [39], neuroblastoma [43], brain tumors [39, 44], various carcinomas of different origin [39, 45–51], and several types of sarcomas (synovial sarcoma, osteosarcoma, and malignant fibrous histiocytoma) [39, 52–57]. SSX1-5 proteins, except for SSX3, are considered cancer-testis antigens (CTAs) [39, 40, 48, 58].

SSX proteins have a Krüppel-associated box (KRAB) domain with transcriptional repression activity in their N-terminus. Moreover, SSX proteins possess a transcriptional repressor domain (SSXRD) in their C-terminus (155–188 position), and this domain is also present in the SS18-SSX fusion protein (Figure 1) [59]. Interestingly, Dimitriadis et al. [60] reported a single SS case with an unusual fusion transcript lacking the SSXRD domain, which may indicate a more significant role of the SS18 protein in SS18-SSX-associated oncogenesis.

There have been several reports concerning the interactions of the SSX proteins, predominantly with regulators of transcription and polycomb group (PcG) proteins. Soulez and colleagues [61] demonstrated that the SSX1 and SSX2 proteins colocalize with chromatin, and Kato et al. [35] reported that the C-terminal region of the SSX1 protein binds to the core histones. Cronwright and coworkers [62] reported that SSX1-9 proteins participate in cell migration, indicating that these proteins may have an analogical role in cancer metastasis. The interactions of the SSX proteins are summarized in Table 1. Interestingly, the RAB3IP and SSX2IP proteins interact with the N-terminal moiety of the SSX2 protein, which is not present in the SS18-SSX fusion protein [63].

## 3. SS18-SSX Fusion Types in Synovial Sarcoma

The SS18-SSX chimeric proteins consist of all but 8 C-terminal amino acids of the SS18 protein and 78 C-terminal amino acid residues of either the SSX1 or SSX2 protein [64]. The *SS18-SSX1* translocation is observed in approximately two-thirds of tumors, and the *SS18-SSX2* variant is found in the remaining cases [10, 65, 66]. In addition, rare cases of SS18-SSX4 chimeric variants in SS have been described, but these have been characterized by high breakpoint variability, with a possible functional unpredictability as a consequence. [67–69]. The rare SS cases lacking the classical *SS18-SSX* fusion gene may represent tumors with unusual variant transcripts, which failed to be detected using conventional approaches [70].

The *SS18-SSX1* fusion type is mostly associated with biphasic SS and the *SS18-SSX2* type strongly correlates with monophasic histology [10, 65, 66, 71, 72]. Saito et al. [73] demonstrated a potential mechanism of such differentiation involving Snail and Slug—the repressors of E-cadherin (CDH1)—which is a crucial determinant of the epithelial phenotype. The loss of E-cadherin has an important role in the epithelial to mesenchymal transition in cancer [74, 75]. The study of Saito and colleagues [73] shows that SS18-SSX2 fusion protein interacts preferentially with Slug and SS18-SSX1 fusion protein interacts preferentially with Snail, which is the stronger repressor of the E-cadherin promoter. These observations provide a simplified explanation for the heterogeneity in the acquisition of epithelial characteristics in SS carrying different fusion types.

Both SS18 and SSX proteins lack DNA-binding domains. The most likely mechanism of their function on both a single and fusion protein level is based on the regulation of transcription and on the direct or indirect protein/protein interactions [8]. According to the individual effects of the SS18 and SSX proteins, the SS18-SSX fusion protein may have both transcriptional activation and repression functions [30].

It was long unknown if the presence of the SS18-SSX fusion protein is the only prerequisite for SS formation. A hypothesis has been proposed stating that SS may develop as a result of a series of molecular interactions in which the activity of the SS18-SSX oncoprotein is only a link in a sequence of other events. To elucidate this possible mechanism, Haldar and coworkers [4] created a mouse model of SS by introducing conditional expression of the *SS18-SSX2* fusion gene in myoblasts. The authors described tumor development in mice that resembled human SS with regard to histological appearance, immunohistochemistry, transcriptional profile, and *SS18-SSX2* fusion gene expression in tumor cells. Their findings supported the idea of *SS18-SSX* translocation as a crucial factor in SS pathogenesis.

Ishida and colleagues [76] demonstrated that SS18-SSX1 can form homooligomers via the QPGY domain similarly to the SS18 protein [31]. Moreover, the SS18-SSX1 fusion protein can form heterooligomers with normal SS18 proteins. Based on these findings, a model of SS has been proposed in which target genes controlled by SS18 homooligomers are repressed by the dominant negative function of the SS18-SSX1/SS18 heterooligomers [76].

Several groups have investigated the prognostic value of the different fusion types, with contradicting results. Some studies reported more favorable outcomes in patients with the *SS18-SSX2* fusion type [10, 65, 71, 77–79], and others failed to find any significant correlation between fusion type and clinical outcome [66, 80, 81].

## 4. Possible Mechanisms in SS Tumorigenesis

Considering published studies, which present downstream and intermediate targets of SS18-SSX1 and SS18-SSX2 fusion oncoproteins, several molecular pathways in SS development may be proposed. Among the possible mechanisms involved in SS, especially the promotion of cell growth and proliferation, deregulation of the TP53 signaling pathway, and interference of chromatin remodeling should be taken into account.

There is also a single study suggesting that the SS-associated fusion protein causes fundamental changes in the cellular cytoskeleton architecture.

**Figure 2:**
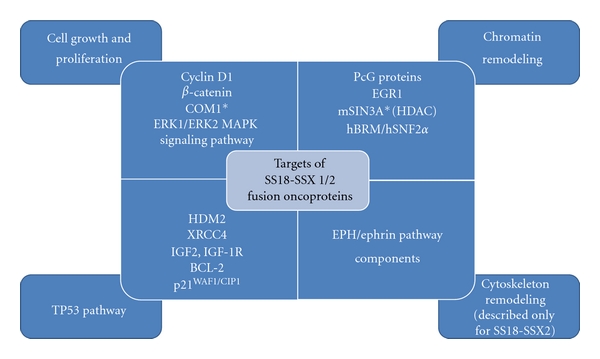
Targets of SS18-SSX1/2 fusion oncoproteins involved in cell growth and proliferation, TP53 pathway, chromatin remodeling, and cytoskeleton remodeling, which may contribute to synovial sarcoma tumorigenesis. COM1-candidate of metastasis protein 1; PcG-polycomb group proteins; EGR-early growth response protein 1; mSIN3A-transcriptional regulator; HDAC-histone deacetylase complex; hBRM/hSNF2*α*-chromatin remodeling complex; TP53-tumor protein p53; HDM2-Mdm2 p53 binding protein homolog (mouse); XRCC4-X-ray repair complementing defective repair in Chinese hamster cells 4; IGF2-insulin-like growth factor 2; IGF-1R-insulin-like growth factor 1 receptor; BCL-2-B-cell CLL/lymphoma 2. *Only COM1 and mSIN3A have been described to interact directly with SS18-SSX1/2 fusion proteins.

Figure 2 summarizes the possible influence of SS18-SSX fusion proteins on described direct and indirect targets, which may contribute to SS tumorigenesis.

## 5. SS18-SSX Fusion Proteins Affect Cell Growth and Proliferation

Several reports indicate cyclin D1, *β*-catenin, TP53 pathway components, early growth response protein 1 (EGR1), and insulin-like growth factor 2 (IGF2) together with its receptor IGF-1R as the most important targets of SS18-SSX fusion oncoproteins, involved in the regulation of cell growth and proliferation.

### 5.1. Cyclin D1 and *β*-Catenin

A study conducted by Xie and colleagues [82] focused on the influence of the SS18-SSX1 and SS18-SSX2 fusion proteins on tumor-relevant and growth-regulatory proteins, including cyclins A/D1/E, cyclin-dependent kinases (CDKs), p27, and *BCL-*2 (a proto-oncogene). *BCL-2 *was expressed at a high level in association with both fusion types. Specifically, SS18-SSX1 was associated with the upregulated expression of cyclins A and D1, and these observations were confirmed by Peng et al. [83]. Upregulation of cyclin D1 has been also associated with SS18-SSX2 fusion type in another study conducted by Xie and colleagues [84]. They suggested that the SS18-SSX2 protein interferes with the ubiquitin-dependent degradation pathway, which results in the upregulation of cyclin D1. An association between the expression level of cyclin D1 and the SS18-SSX2 fusion protein was also investigated by Törnkvist and coworkers [85], who found that the SS18-SSX2 protein is responsible for the maintenance of both cyclin D1 expression and cell proliferation in the examined SS cell lines.

Pretto et al. [86] suggested that *β*-catenin is another downstream target of the SS-associated fusion proteins, however they tested only the SS18-SSX2 variant. *β*-catenin is an adherent junction-associated protein which connects cadherins to the cytoskeleton. Alternatively, when localized in the nucleus, *β*-catenin is an essential effector of the WNT signaling pathway. The misregulation of *β*-catenin-mediated signaling leads to the development of numerous human malignancies [87]. Pretto et al. [86] demonstrated that *SS18-SSX2* stimulates *β*-catenin signaling in a P300-dependent manner. The fusion transcript recruits *β*-catenin to the nucleus, forming a transcriptionally active nuclear complex. Furthermore, the inhibition of *SS18-SSX2* expression contributes to the loss of the nuclear *β*-catenin signal and a strong decrease of *β*-catenin signaling activity. These findings suggest that *SS18-SSX2* triggers tumor development partially through the *β*-catenin signaling but it should be further investigated whether SS18-SSX1 has the same effect.

Interestingly, Horvai and colleagues [88] associated the activation of nuclear *β*-catenin with the upregulation of cyclin D1 both in primary and metastatic SS tissue specimens with confirmed *t*(*X*;18) translocation. A probable mechanism for this upregulation engages *β*-catenin and the WNT signaling pathway, which activate the transcription complex involving TCF/LEF. As a result, *β*-catenin contributes to the increased cyclin D1 level, which is characteristic of many cancers. These findings agree with the study by Bozzi and coworkers [89] who examined 17 cases of SS in children and adolescents with the confirmed *SS18-SSX* fusion (11 cases with *SS18-SSX1* and 6 cases with *SS18-SSX2*). They observed nuclear *β*-catenin localization in monophasic SS specimens and cyclin D1 overexpression in monophasic and biphasic SS specimens.

### 5.2. TP53 Pathway

Xie et al. [90] applied the antisense strategy to block the expression of the *SS18-SSX2* fusion gene in SS cells, and they reported that the expression of X-ray repair complementing defective repair in Chinese hamster cells 4 (*XRCC4*) is significantly decreased in such conditions. The unfixed DNA damage in SS may activate the TP53 checkpoint pathway to induce growth arrest or apoptosis. This experiment has been conducted only in one SS cell line carrying SS18-SSX2 transcript, but two other studies of D'Arcy and coworkers demonstrated that TP53 is affected in cell lines expressing both *SS18-SSX1* and *SS18-SSX2*. Firstly, they demonstrated that SS18-SSX1 promotes TP53 ubiquitination and degradation by a mechanism involving the HDM2 (Mdm2 p53 binding protein homolog in mouse). The fusion protein inhibited autoubiquitination and increased the half-life of HDM2, making it more stable in cells [91]. A subsequent study confirmed that TP53 is specifically affected by HDM2 also in SYO-1 cell line, derived from biphasic SS and carrying SS18-SSX2, which presented the increase of TP53 stability, activation of TP53 target genes, growth arrest, and apoptosis upon treatment with HDM2 antagonist [92]. Another observation [91] was that SS18-SSX1 attenuates the TP53 response and changes the expression levels of several TP53-regulated genes, such as *BBC3* (BCL2 binding component 3) and *PMAIP1* (phorbol-12-myristate-13-acetate-induced protein 1), but it does not affect the expression level of the p21^WAF1/CIP1^ (also known as *CDKN1A*-cyclin-dependent kinase inhibitor 1A). However, contradictory results have been published by Tsuda and coworkers [93] who reported that SS18-SSX1 upregulates p21^WAF1/CIP1^ protein in the manner independent of the hBRM chromatin remodeling factor and TP53 but dependent on the SP1/SP3 transcription factors.

### 5.3. EGR1

Lubieniecka and coworkers [94] reported that the SS18-SSX2 fusion protein inhibits a cancer-related gene *EGR1* through repressive histone modifications and recruitment of PcG proteins. *EGR1* encodes a serum-inducible zinc finger protein, which is crucial for cell proliferation, differentiation and apoptosis. The same group demonstrated that *EGR1* is a target of the fusion protein but they only examined two cell lines carrying *SS18-SSX2 *fusion variant. No cell line expressing *SS18-SSX1* fusion gene has been included in the study. The downregulation of *EGR1 *in SS has also been observed in cDNA microarray experiments [94, 95]. Moreover, Su et al. [96] demonstrated that EGR1 affects the expression of phosphatase and tensin homolog deleted in chromosome 10 (*PTEN*), which is a tumor suppressor gene and an important phosphatase that regulates the PI3K/AKT survival pathway. Both *EGR1* and *PTEN* have been reported to be downregulated by the specific microRNA, miR-183, and the elevated level of miR-183 has been observed in SS and other tumor types [97]. These data suggest the important role of the *EGR1* and *PTEN* tumor suppressors in SS oncogenesis.

### 5.4. *IGF2* and *IGF-1R*

*IGF2* is another target of the SS18-SSX fusion proteins. Upregulation of this gene has been associated with the expression of both SS18-SSX1 and SS18-SSX2 fusion proteins [95, 98, 99]. Upregulation of *IGF2* is necessary for tumor formation *in vivo* and for the proliferation of cultured SS cells [99, 100]. The antiapoptotic and mitogenic function of IGF2 is mediated by IGF-1R, and the expression of this receptor has been also demonstrated in SS cell lines and tumor specimens bearing SS18-SSX1 and SS18-SSX2 fusion proteins [85, 100–102]. In addition, Friedrichs et al. [100] reported that also AKT and p44/42 MAPK (elements of IGF-1R-related signaling pathways) were activated in the analyzed tumor specimens. Although *IGF2* has an important role in SS, the overexpression of *IGF2* is not exclusive to this sarcoma subtype. For example, several expression profiling studies have demonstrated *IGF2* overexpression as a characteristic feature of gastrointestinal stromal tumors (GISTs) and rhabdomyosarcomas [103–106].

The cell proliferation regulator, candidate of metastasis 1 (*COM1*), also known as *p8* or nuclear protein transcriptional regulator 1 (*NUPR1*), and ERK1/ERK2 MAPK signaling pathway components have been proposed as other possible targets of SS18-SSX fusion proteins, involved in the cell proliferation regulation. However, there are only single reports describing their role in SS.

Ishida et al. [76] observed that both SS18-SSX1/2 fusion oncoproteins directly downregulate the expression of *COM1, *which is expressed at low level both in cell lines carrying different types of translocation, as well as in SS tumor specimens. The authors demonstrated also that the knockdown of *SS18-SSX2* results in a strong upregulation of *COM1* expression, which reduces *in vitro* cell growth and colony formation of SYO-1 cell line. Additionally, the restoration of COM1 expression induced apoptosis in this cell line. Overall, their results indicated that the maintenance of COM1 expression at a low level has an important role in SS growth.

Cai et al. [107] performed RNA interference experiments targeting the 19-nucleotide sequence of SS18-SSX2 mRNA in SYO-1 cells, demonstrating that downregulation of the fusion gene inhibits cell proliferation and diminishes the protein levels of ERK1, ERK2, p-ERK, and cyclin D1.

## 6. SS18-SSX Fusion Proteins Are Involved in Chromatin Remodeling

PcG proteins, histone deacetylase complex (HDAC), and hBRM/hSNF2*α* complex are the most important targets of SS18-SSX fusion oncoproteins involved in chromatin remodeling.

### 6.1. Polycomb Group Proteins

PcG proteins are involved in the repression of gene transcription through the modulation of chromatin structure. Functional deregulation of polycomb complexes has been previously demonstrated during cancer progression. The polycomb complex is usually involved in tumorigenesis as a result of its upregulation [108–110]. However, downregulation of the polycomb complex has been described in germ cell tumors [111] and some breast tumors [112].

According to the study of dos Santos and colleagues [113], SSX2 and both SS18-SSX1/2 proteins colocalize with PcG proteins. Specifically, co-localization of SS18-SSX1 and SS18-SSX2 fusion proteins with RING1 proteins, SS18-SSX2 fusion proteins with HPC2 proteins, and SSX2 proteins with RING1 proteins, BMI1 proteins and chromatin have been reported. However, these colocalizations are not the result of direct physical interactions. These findings were consistent with a study by Soulez et al. [61] who demonstrated that SSX1, SSX2, SS18-SSX1, and SS18-SSX2 proteins colocalize with BMI1 and that SSX1, SSX2, and SS18-SSX2 proteins colocalize with chromatin. Moreover, colocalization of SS18-SSX2 fusion proteins with RING1 proteins has also been observed. Yet, Western blot analysis did not confirm the direct interactions of these proteins [61]. Furthermore, polycomb deregulation by SS18-SSX fusion proteins has been demonstrated in two other studies. Cironi and colleagues [98] showed that the polycomb target genes appear to be affected by the expression of *SS18-SSX1*. Barco et al. [114] reported that the SS18-SSX2 fusion protein modulates the silencing activity of the polycomb repressive complex and destabilizes BMI1 proteins, leading to the impairment of polycomb-dependent histone H2A ubiquitination and reactivation of the polycomb target genes.

### 6.2. Histone Deacetylase Complex (HDAC)

Ito and colleagues [34] observed that SS18 and SS18-SSX1 proteins (but not SS18-SSX2 protein) can bind mSIN3A proteins, which are a part of the histone deacetylase complex (HDAC). HDAC affects gene expression by acting on chromatin through transcriptional factors, corepressors and methyl-CpG binding proteins. Based on these findings, Ito et al. [115] further investigated the impact of the histone deacetylase inhibitor, FK228, on SS growth both *in vitro* and *in vivo*. They examined cell lines carrying both the SS18-SSX1 and SS18-SSX2 fusion proteins. As a result of treatment with the FK228 inhibitor, the growth of cultured SS cells and SS xenografts in mice was significantly reduced. Also tumor weight and tumor density decreased after FK228 treatment. Remarkably, cells carrying the SS18-SSX2 fusion protein were more sensitive than those carrying the SS18-SSX1 one.

### 6.3. hBRM/hSNF2*α* Complex

Nagai and coworkers [116] assessed the transforming activity of the SS18-SSX1 fusion protein, and they correlated the growth rate of the cells with the expression level of the *SS18*, *SSX1*, and *SS18-SSX1* genes introduced into three independent clones of 3Y1 rat fibroblast cell lines. 3Y1 cells expressing *SS18-SSX2* fusion gene were not included in the study. The researchers reported that the human *SS18*-*SSX1* fusion gene imposed the highest growth rate among the genes tested. Furthermore, they established the association between the SS18-SSX1 protein and the chromatin remodeling complex, hBRM/hSNF2*α*. They proposed that this complex has an essential role in SS18-SSX1-induced transformation, suggesting that it may downregulate the deleted in colorectal carcinoma (DCC) tumor suppressor.

## 7. SS18-SSX2 Fusion Protein and Its Role in Cytoskeleton Remodeling

Barco et al. [117] presented another possible mechanism of carcinogenesis triggered by the SS18-SSX2 fusion oncoprotein. They reported that SS18-SSX2 stabilizes the microtubule network and stimulates the expression and activation of EPH/ephrin pathway components. The EPH/ephrin system is involved in the regulation of cytoarchitecture and determination of cell position during development and tissue regeneration. In tumors, the EPH/ephrin system has been linked to angiogenesis, loss of cell adhesion, and enhanced migration. Overall, the study suggested that the SS18-SSX2 fusion protein causes fundamental changes in the cellular cytoskeleton architecture; however, this mechanism has not been investigated in cells carrying SS18-SSX1 fusion transcript.

## 8. Discussion

### 8.1. Development of Targeted Therapy for Synovial Sarcoma

Based on the assumption that cancer-related pathways and proteins that are upregulated in SS may serve as possible therapeutic targets, a few important directions should be considered. Bozzi and colleagues [89] showed that EGFR and platelet-derived growth factor receptors (PDGFRA and PDGFRB) are upregulated in SS with subsequent PI3K/AKT/mTOR pathway activation. Consecutive reports by Dobashi et al. [118] and Friedrichs et al. [119] also demonstrated the activation of the PI3K/AKT/mTOR pathway in SS. These reports suggest that inhibitors of the PI3K/AKT/mTOR pathway may be used for a potential therapy of SS through the repression of multiple upstream targets of this pathway. Several other studies have confirmed the upregulation of the receptor tyrosine kinases EGFR, PDGFRA and PDGFRB in SS that can be targeted by specific small molecule inhibitors [120–127]. HER-2/neu, BCL-2, and fibroblast growth factor receptor 2 (FGFR2) are among other proteins considered as potential targets in SS [6, 17, 82, 122, 128]. Additionally, IGF-1R may be a suitable target in SS therapy, and therapeutic antibodies against IGF-1R and small molecules inhibiting the tyrosine kinase activity of IGF-1R have been already developed [85, 101, 129].

Since the presence of SS18-SSX fusion proteins is a proven prerequisite for SS oncogenesis, a detailed insight into the mechanisms of their interactions may contribute to the development of targeted therapies, which are still unavailable for SS patients. Several direct effectors of SS18-SSX fusion proteins in SS have been already confirmed but they have not yet been examined as potential therapeutic targets. Thus, it may be reasonable to undertake new studies with the following aims: (1) to accurately characterize the nature of these interactions, including the domains or amino acid residues involved; (2) to screen small molecule libraries to detect potential molecules that interrupt selected interactions; (3) to test selected small molecules in synovial sarcoma animal models; (4) to transfer these results to clinical trials. Such strategy is reasonable especially in the light of recent findings made by Erkizan and coworkers [130] who identified a small molecule inhibitor of *EWS-FLI1* fusion transcript interaction with RNA helicase A (RHA), which is important in the oncogenesis of Ewing's sarcoma family tumors (ESFTs).

Moreover, the SS18 and SSX proteins have been described to individually interact with several important regulators of transcription. Hence, it is rational to test in the future studies whether the SS18-SSX fusion proteins also interact with these molecules. Among proteins that colocalize both with SSX and SS18-SSX are PcG proteins, which appear to be an interesting target in synovial sarcoma therapy. The important role of PcG proteins in oncogenesis has been already extensively reviewed [131, 132] and some recent publications support their use as molecular targets in anticancer therapy. Dimri and coworkers [133] showed that the aberrant expression of PcG protein enhancer of zeste homologue 2 (EZH2) can be suppressed by dietary omega-3 polyunsaturated fatty acids (PUFAs), leading to decrease in the invasion potential of breast cancer cells. Another study published by Kemp and colleagues [134] demonstrated that pharmacological inhibition of polycomb repressor complex-2 (PRC-2) is a successful therapeutic strategy in malignant pleural mesothelioma. Even though RING1 and BMI1 proteins which colocalize with SSX and SS18-SSX are the components of another polycomb repressor complex-1 (PRC-1), the abovementioned results provide rationale to test the influence of PRC-1 inhibition on the synovial sarcoma oncogenesis.

As of yet, the completed clinical trials investigating the role of selected targets in SS did not point to any successful therapeutic strategy. A phase II study conducted by the European Organization for Research and Treatment of Cancer (EORTC) Soft Tissue and Sarcoma Group demonstrated that gefitinib treatment specifically targeting EGFR failed in SS patients [135]. The reports about EGFR upregulation in SS have not indicated any direct interaction or association with the SS18-SSX1 or SS18-SSX2 fusion proteins which might explain why inhibition of EGFR pathway was not successful in SS treatment [122, 126]. However, gefitinib was the only EGFR inhibitor examined thus far in SS and the efficacy of other small molecule EGFR inhibitors remain to be explored. Similarly in reference to the upregulation of PDGFRA/B in SS, a phase II study assessing imatinib efficacy in 10 sarcoma subtypes determined that it is not an active agent in SS [136]. Nonetheless, another phase Ib/II clinical trial has been undertaken to examine the efficacy of imatinib in combination with mTOR inhibitor everolimus in SS patients (NCT01281865). Another ongoing phase II study including SS patients aims to evaluate the combined use of mTOR inhibitor temsirolimus with IGF-1R monoclonal antibody cixutumumab (NCT01016015). Furthermore, there are two phase II clinical trials in progress including SS patients, which examine the treatment with HDAC inhibitors (NCT01112384 and NCT01136499). Hopefully the results of these trials will shed more light on possible directions in SS targeted therapy.

### 8.2. Hypothetical Network of Interactions

Interestingly, there have been several reports demonstrating interactions between the various genes and proteins mentioned in this review without reference to SS development or SS maintenance. Many of the SS18-SSX1/2 targets, such as COM1, HDM2, cyclin D1, and EGR1, have been described to interact with P300 proteins [137–141]. In addition, HDM2 may interact with p21^WAF1/CIP1^ and may associate with chromatin [142]. Furthermore, the nuclear epidermal growth factor receptor (EGFR) participates in cyclin D1 transcription [143], and cyclins D1 and A bind to p21^WAF1/CIP1^ [144]. Tsuda et al. [93] described that the upregulation of p21^WAF1/CIP1^ is dependent on the SP1/SP3 transcription factors. Importantly, the SP1 transcription factor interacts with EGR1, HDM2 and TP53, which are all implicated in SS tumorigenesis via SS18-SSX1/2 fusion proteins [145–150]. Further studies of these potential interactions in the context of SS should be conducted; they may provide a broader perspective on oncogenic pathways in SS.

### 8.3. SS18-SSX1 versus SS18-SSX2

As far as pathways involving cell growth, cell proliferation, TP53, and chromatin remodeling are concerned, it has been shown that both the SS18-SSX1 and SS18-SSX2 fusion proteins may interfere with them, but often through distinct molecular interactions. The cytoskeleton remodeling mechanism has been examined exclusively for the SS18-SSX2 fusion type. It should be taken into consideration that there has been still a relatively small number of studies conducted to determine downstream and intermediate targets of SS18-SSX1/2 fusion oncoproteins. Probably many of their interactions and direct effects remain unknown.

Notably, it is important to distinguish between the SS18-SSX1 and SS18-SSX2 oncoproteins while studying the role of the fusion transcript in SS formation. Both by functional and expression profiling studies, it has been shown that these proteins may have distinct molecular activities even though their sequences are highly homologous and they induce tumors with similar pathological features. Inagaki et al. [77] compared the impact of *SS18-SSX1* and *SS18-SSX2* fusion types on the expression level of several tumor cell proliferation-associated genes and other tumor-related pathological parameters in SS primary tumor specimens. As compared to the SS18-SSX2 fusion protein, the SS18-SSX1 fusion protein is related to higher mitotic rate and higher proliferation index as measured by Ki-67 staining. In turn, Fernebro and coworkers [11] conducted gene expression profiling studies to determine gene expression differences between *SS18-SSX1* and *SS18-SSX2* SS variants. Among the upregulated genes associated with the SS18-SSX1 fusion protein they listed genes involved in oncogenesis, such as *TCF7*, *ZIC2*, *IGFBP3*, *SPAG7*, *AGRN*, *VIL2*, *AXL*, *RALGDS*, and *CDC2LI*, in addition to genes encoding metallothioneins, histones and G protein-coupled receptors. Genes characteristically overexpressed in SS with the SS18-SSX2 fusion type include *FOXC1*, *GAS1*, and *NCAM1*.

Because SS specimens carrying different SS18-SSX fusion proteins may have distinct molecular characteristics, it seems inappropriate to formulate conclusions about the function of the SS18-SSX fusion protein based on the results obtained only for one fusion type. Moreover, some reports describe a general role of the SS18-SSX fusion protein even though the actual fusion type was not defined or may have been only presumed from the SS cell line used in the particular experiment. In the search for the functions of SS18-SSX1 and SS18-SSX2, it is necessary to conduct studies that include cell lines or tumor specimen groups with different types of fusion proteins, allowing their possible heterogeneous molecular functions to be recognized.

Current state of the SS research indicates that SS18-SSX fusion proteins deregulate mostly cell growth, cell proliferation, TP53 pathway, and chromatin remodeling mechanisms. Several molecules involved in these pathways occur to be emerging therapeutic targets, especially those directly interacting with SS-associated fusion proteins. It is crucial for the prospective clinical trials to select potential therapeutic targets on the basis of functional studies that explore the critical drivers of SS oncogenesis. However, many aspects of SS tumorigenesis and network of SS18-SSX fusion proteins interactions remain to be elucidated.

## Figures and Tables

**Table 1:** Interactors of SSX proteins.

Gene symbol	Gene description	Function	Reference
	SSX1		

*FUBP3*	Far upstream element (FUSE) binding protein 3	Regulation of transcription	[151]
*BMI1*	BMI1 polycomb ring finger oncogene	PcG protein-transcriptional repressor	[61]
*RING1A*	Ring finger protein 1	PcG protein-transcriptional repressor	[61]
*LHX4*	LIM homeobox 4	Transcription factor	[152]

	SSX2		

*BMI1*	BMI1 polycomb ring finger oncogene	PcG protein-transcriptional repressor	[61]
*RING1A*	Ring finger protein 1	PcG protein-transcriptional repressor	[61]
*RING1B (RNF2)*	Ring finger protein 2	PcG protein-transcriptional repressor	[117]
*RAB3IP*	RAB3A interacting protein (rabin3)	Protein transport	[63, 153]
*SSX2IP*	Synovial sarcoma, X breakpoint 2 interacting protein	Cell adhesion	[63, 153]

	SSX3		

*DDIT3 (CHOP)*	DNA-damage-inducible transcript 3	Transcription factor	[151]
*ZBTB25*	Zinc finger and BTB domain containing 25	Regulation of transcription	[151]
*ZBTB3*	Zinc finger and BTB domain containing 3	Regulation of transcription	[151]
*PCBD2*	Pterin-4 alpha-carbinolamine dehydratase/dimerization cofactor of hepatocyte nuclear factor 1 alpha (TCF1) 2	Regulation of transcription	[151]
*ZNF496*	Zinc finger protein 496	Regulation of transcription	[151]
*ZSCAN1*	Zinc finger and SCAN domain containing 1	Regulation of transcription	[151]
*SSX2IP*	Synovial sarcoma, X breakpoint 2 interacting protein	Cell adhesion	[63, 153]

	SSX4		

*HIF1A*	Hypoxia inducible factor 1, alpha subunit	Transcription factor	[151, 153]
*PAX9*	Paired box 9	Transcription factor	[151]
*XBP1*	X-box binding protein 1	Transcription factor	[151]
*LZTR1*	Leucine-zipper-like transcription regulator 1	Transcription factor	[151]

	SSX5		

*NFE2*	Nuclear factor (erythroid-derived 2), 45 kDa	Regulation of transcription	[151]
*PCBD2*	Pterin-4 alpha-carbinolamine dehydratase/dimerization cofactor of hepatocyte nuclear factor 1 alpha (TCF1) 2	Regulation of transcription	[151]
*ZSCAN1*	Zinc finger and SCAN domain containing 1	Regulation of transcription	[151]
